# Expression of leukotriene B_4_ receptor 1 defines functionally distinct DCs that control allergic skin inflammation

**DOI:** 10.1038/s41423-020-00559-7

**Published:** 2020-10-09

**Authors:** Tomoaki Koga, Fumiyuki Sasaki, Kazuko Saeki, Soken Tsuchiya, Toshiaki Okuno, Mai Ohba, Takako Ichiki, Satoshi Iwamoto, Hirotsugu Uzawa, Keiko Kitajima, Chikara Meno, Eri Nakamura, Norihiro Tada, Yoshinori Fukui, Junichi Kikuta, Masaru Ishii, Yukihiko Sugimoto, Mitsuyoshi Nakao, Takehiko Yokomizo

**Affiliations:** 1 Department of Biochemistry, Juntendo University Graduate School of Medicine, Tokyo, 113-8421 Japan; 2 Department of Medical Cell Biology, Institute of Molecular Embryology and Genetics, Kumamoto University, Kumamoto, 860-0811 Japan; 3 Department of Cell Signaling, Graduate School of Medical and Dental Sciences, Tokyo Medical and Dental University, Tokyo, 113-8510 Japan; 4 Department of Pharmaceutical Biochemistry, Graduate School of Pharmaceutical Sciences, Kumamoto University, Kumamoto, 862-0973 Japan; 5 Department of Developmental Biology, Graduate School of Medical Sciences, Kyushu University, Fukuoka, 812-8582 Japan; 6 Laboratory of Genome Research, Research Institute for Diseases of Old Age, Juntendo University Graduate School of Medicine, Tokyo, 113-8421 Japan; 7 Division of Immunogenetics, Department of Immunobiology and Neuroscience, Medical Institute of Bioregulation, Kyushu University, Fukuoka, 812-8582 Japan; 8 Department of Immunology and Cell Biology, Graduate School of Medicine and Frontier Biosciences, Osaka University, Osaka, 565-0871 Japan

**Keywords:** LTB4, BLT1, dendritic cells, inflammation, lipid mediator, Gene regulation in immune cells, Inflammation

## Abstract

Leukotriene B_4_ (LTB_4_) receptor 1 (BLT1) is a chemotactic G protein-coupled receptor expressed by leukocytes, such as granulocytes, macrophages, and activated T cells. Although there is growing evidence that BLT1 plays crucial roles in immune responses, its role in dendritic cells remains largely unknown. Here, we identified novel DC subsets defined by the expression of BLT1, namely, BLT1^hi^ and BLT1^lo^ DCs. We also found that BLT1^hi^ and BLT1^lo^ DCs differentially migrated toward LTB_4_ and CCL21, a lymph node-homing chemoattractant, respectively. By generating LTB_4_-producing enzyme LTA_4_H knockout mice and CD11c promoter-driven Cre recombinase-expressing BLT1 conditional knockout (BLT1 cKO) mice, we showed that the migration of BLT1^hi^ DCs exacerbated allergic contact dermatitis. Comprehensive transcriptome analysis revealed that BLT1^hi^ DCs preferentially induced Th1 differentiation by upregulating IL-12p35 expression, whereas BLT1^lo^ DCs accelerated T cell proliferation by producing IL-2. Collectively, the data reveal an unexpected role for BLT1 as a novel DC subset marker and provide novel insights into the role of the LTB_4_-BLT1 axis in the spatiotemporal regulation of distinct DC subsets.

## Introduction

Dendritic cells (DCs) are specialized antigen-presenting cells that reside at host-environment boundaries, such as the skin, lungs, and intestine. DCs capture antigens in the periphery and migrate toward draining lymph nodes. Migrated DCs activate naïve T cells by presenting antigen-loaded MHC class II molecules and by producing cytokines that induce T cell differentiation (i.e., interleukin [IL]-12 for Th1 cells; IL-4 for Th2 cells; IL-6, IL-23, and transforming growth factor [TGF]-β for Th17 cells; and TGF-β for regulatory T cells [Tregs]). Thus, DCs are a crucial “control tower” for acquired immune responses. In addition, recent reports show that in addition to migrating to lymph nodes, DCs migrate toward peripheral inflammatory areas to form DC-T cell clusters; both pathways are important for efficient antigen presentation and T cell expansion.^1–4^ Thus, the migratory and cytokine-producing abilities of DCs are crucial for efficient control of acquired immune responses.

Acquired immunity is mediated by cytokines, chemokines, noncoding RNAs, and lipid mediators. Growing evidence suggests that among these factors, lipid mediators are involved in accelerating and regulating immunological responses. A classical lipid mediator, prostaglandin E_2_ (PGE_2_), and its receptor, EP4, promote immune inflammation associated with contact hypersensitivity and experimental autoimmune encephalomyelitis (EAE) by inducing the differentiation and expansion of Th1 and Th17 cells.^5,6^ Another lipid mediator, sphingosine 1 phosphate (S1P), is a lysophospholipid that attracts activated T cells in the lymph nodes to efferent lymphatic vessels, thereby amplifying acquired immune responses. Indeed, FTY720 (fingolimod), a functional antagonist of the S1P receptor S1P_1_, is used as a drug to treat autoimmune diseases such as multiple sclerosis because it inhibits the S1P-S1P_1_-dependent egress of lymphocytes from the lymph nodes and reduces the recirculation of autoaggressive T cells.^7–12^ An alternative proresolution lipid mediator, resolvin E1 (RvE1), inhibits DC migration to the skin and attenuates contact dermatitis.^13^ Furthermore, phospholipase A2 group IID resolves contact hypersensitivity by driving the production of anti-inflammatory lipid mediators, including RvD1 and 15-deoxy-Δ^12,14^-prostaglandin J_2_.^14^ Advances in lipid detection methods (e.g., LC-MS/MS) have led to the discovery of more than 100,000 species of lipids within the human body; however, the functions of lipids during acquired immune responses remain largely unknown.

**Fig. 1:**
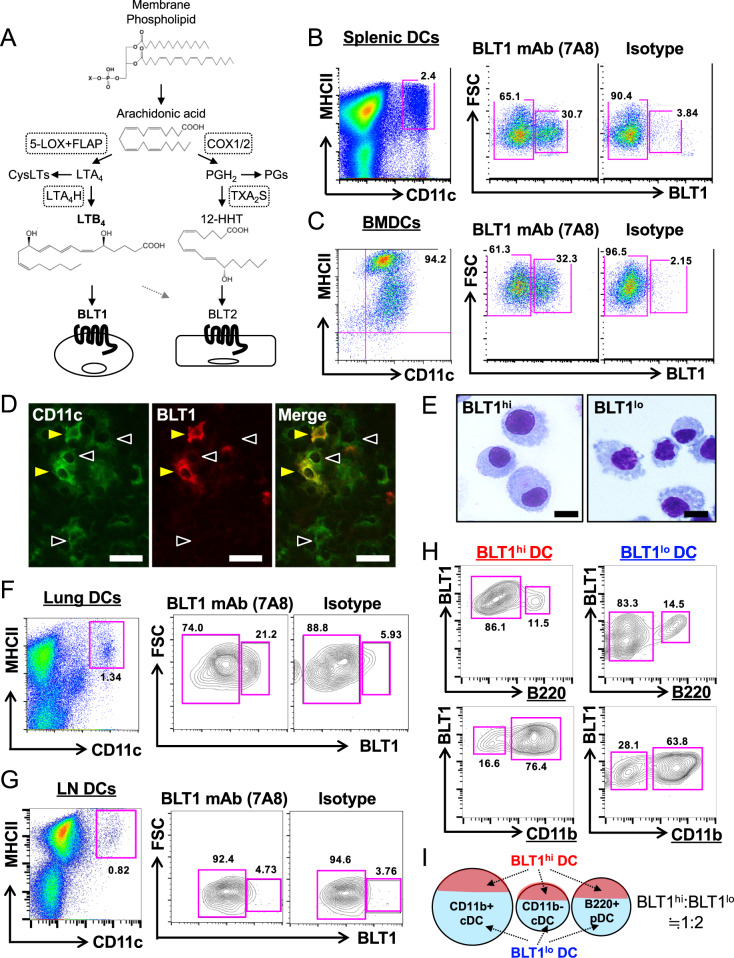
BLT1-positive and BLT1-negative DC subsets. **a** Schematic representation of LTB_4_ production. Arachidonic acid released from membrane phospholipids is converted into LTA_4_ by the enzymes 5-LOX and FLAP. LTB_4_ is generated from LTA_4_ by the enzyme LTA_4_H. LTB_4_ acts as an endogenous ligand for the BLT1 receptor. LTA_4_ is also converted into CysLTs (LTC_4_, LTD_4_, and LTE_4_). **b** Splenocytes were stained with antibodies specific for CD11c, MHC class II, and mouse BLT1. CD11c+/MHC class II+DCs were analyzed using an anti-mouse BLT1 monoclonal antibody (7A8). **c** BMDCs were stained with antibodies specific for CD11c, MHC class II, and BLT1. **d** Immunofluorescence staining of the mouse spleen was performed using anti-CD11c and anti-mouse BLT1 antibodies. Closed arrowheads indicate BLT1-positive DCs; open arrowheads indicate BLT1-negative DCs. Bars, 20 μm. **e** CD11c+/BLT1+BLT1^hi^ DCs and CD11c+/BLT1- BLT1^lo^ DCs derived from BMDCs were sorted and stained with a Diff-Quik solution. Bars, 10 μm. DCs from the lungs (**f**) and popliteal lymph nodes (**g**) were stained with antibodies specific for CD11c, MHC class II, and BLT1 and then subjected to flow cytometric analysis. **h** Splenic BLT1^hi^ and BLT1^lo^ DCs were stained with antibodies specific for B220 and CD11b. The percentage of cells in the violet square is shown. **i** A schematic summary of the corelationships among BLT1^hi^ DCs, BLT1^lo^ DCs, CD11b+cDCs, CD11b- cDCs, and B220+pDCs is shown

Leukotriene B_4_ (LTB_4_) is a classic inflammatory lipid mediator produced by myeloid cells such as granulocytes, macrophages, and DCs.^15–18^ LTB_4_ is generated from arachidonic acid by 5-lipoxygenase (5-LOX), 5-LOX-activating protein (FLAP), and LTA_4_ hydrolase (LTA_4_H). Previously, we cloned BLT1 as a high-affinity receptor for LTB_4_ (Fig. 1a).^19^ BLT1 is a G protein-coupled receptor expressed by neutrophils, eosinophils, macrophages, DCs, and activated T cells and is conserved from zebrafish to mammals.^20–30^ We generated BLT1-deficient mice and used them to show that BLT1 plays detrimental roles in Th1-, Th2-, and Th17-dependent diseases.^26,31,32^ Wang et al. further demonstrated that BLT1 is expressed by CD4+/CD25+/Foxp3+ Tregs and promotes resolution of acute lung injury.^33^ Moreover, Goodarzi et al. showed that BLT1 expression is greatly induced in CD8+ T cells during differentiation into effector cells and promotes T cell recruitment to inflamed tissues.^29^ Thus, BLT1 acts as a crucial player during acquired immune responses. However, since BLT1 is expressed by various leukocytes, its cell-specific role in acquired immunity remains largely unknown.

Here, we developed an anti-mouse BLT1 monoclonal antibody,^34,35^ LTA_4_H-deficient mice, and CD11c promoter-driven Cre recombinase-expressing BLT1 conditional knockout (cKO) mice and used these tools to examine the role of BLT1 specifically expressed by DCs. The results revealed the immune response-amplifying function of BLT1 expressed by DCs and the presence of novel DC subsets defined by the expression levels of BLT1, namely, BLT1^hi^ and BLT1^lo^ DCs. Furthermore, we show that these DCs play critical immune roles by generating distinct cytokine profiles and exhibiting different migratory activities. Taken together, these findings provide new insights into the DC-specific role of the LTB_4_-BLT1 axis during the immune response and suggest new therapeutic targets for immune-mediated diseases, such as delayed-type allergic contact dermatitis.

## Materials and methods

### Reagents and antibodies

2,4-Dinitrofluorobenzene (DNFB), 2,4-dinitrobenzenesulfonate (DNBS), an ovalbumin (OVA) peptide (323–339), and lipopolysaccharide (LPS) were purchased from Sigma-Aldrich (St. Louis, MO, USA). LTB_4_ and U75302 were purchased from Cayman Chemical (Ann Arbor, MI, USA). CpG DNA was purchased from InvivoGen (San Diego, CA, USA). Murine GM-CSF was purchased from PeproTech (London, UK). CellTracker Green 5-chloromethylfluorescein diacetate (CMFDA) and CellTracker Red CMTPX were purchased from Invitrogen (Carlsbad, CA, USA). A recombinant mouse CCL21 protein and recombinant IL-2 protein were purchased from R&D Systems (Minneapolis, MN, USA). The anti-mouse BLT1 monoclonal antibody 7A8 was generated in-house, and biotinylation was performed using the EZ-link sulfo-NHS-Biotin Kit (Pierce-Thermo Fisher Scientific, Rockford, IL, USA). An anti-mouse BLT1 rabbit polyclonal antibody was generated in-house by immunizing a rabbit with the C-terminal peptide of mouse BLT1 (DSFMTSSTIPESSK). The antibody was affinity-purified using a peptide antigen. A FITC-conjugated anti-CD11c antibody, a phycoerythrin (PE)-conjugated anti-MHC class II antibody, streptavidin (SA)-allophycocyanin (APC), 7-amino actinomycin D (7-AAD), a PE-conjugated anti-CD80 antibody, a PE-conjugated anti-CD86 antibody, a PE-conjugated anti-B220 antibody, a PE-conjugated anti-CD11b antibody, a FITC-conjugated anti-IFN-γ antibody, an anti-IL-12p40 antibody, a PerCP-Cyanine 5.5-conjugated anti-CD4 antibody, biotin-conjugated mouse IgG_1_, and rat IgG_2b_ antibodies were purchased from eBioscience (San Diego, CA, USA). An anti-IL-12p35 antibody was purchased from Invitrogen. An anti-phospho-ERK1/2 antibody and Na^+^-K^+^ ATPase-specific antibodies were purchased from Cell Signaling Technology (Danvers, MA, USA). An anti-ERK2 antibody and anti-β-actin antibody were purchased from Santa Cruz Biotechnology (Santa Cruz, CA, USA).

### Lipid extraction and LC-MS/MS

BMDCs (1 × 10^6^ cells) derived from LTA_4_H WT, heterozygous, or KO mice were stimulated for 30 min with 2 μM A23187, and the culture supernatants were collected. The same amount of ice-cold methanol was added to each sample. Frozen ears from mice with allergic dermatitis were crushed using an SK mill (Tokken Inc., Chiba, Japan), and lipids were extracted by the addition of methanol, followed by centrifugation. LC-MS/MS analyses were performed as described below. Prepared samples were diluted in a dilution solution (water:formic acid [100:0.1, v/v]). An internal standard mixture was then added, and the samples were loaded onto C18 cartridges (Oasis HLB cartridge, Waters, Milford, MA). These cartridges were then washed with a washing solution (water:formic acid [100:0.1, v/v]; water:methanol:formic acid [85:15:0.1, v/v/v]; and petroleum ether:formic acid [100:0.1, v/v]). Fatty acid-enriched samples were eluted into glass vials using 200 μl of methanol containing 0.1% formic acid. The samples were then analyzed by tandem LC-MS/MS. A Prominence HPLC system (Shimadzu, Kyoto, Japan) and TSQ Quantum Ultra triple-stage quadrupole mass spectrometer were connected in tandem. The mobile phases A and B comprised water and acetonitrile with 0.1% formic acid, respectively. Separation and enrichment were performed using a Capcell Pak C18 MGS3 separating column (Shiseido, Tokyo, Japan) and an Opti-Guard Mini C18 trapping column (Optimize Technologies, Oregon City, OR, USA). The conditions used to detect LTB_4_ were as follows: retention time (min), 10.98; precursor ion (m/z), 335; product ion (m/z), 195; and collision energy (V), 16. Data were analyzed using XCalibur 2.1 (Thermo Fisher Scientific).

### DNA microarray analysis

Total RNA was extracted from BLT1^hi^ and BLT1^lo^ DCs (treated with LPS or CpG DNA or left untreated for 4 h) using TRIzol reagents. cDNA was prepared and labeled using the Ambion WT Expression Kit (Affymetrix, San Diego, CA, USA) and GeneChip WT Terminal Labeling Kit (Affymetrix). The labeled samples were subjected to hybridization with the GeneChip Hybridization Wash, Stain Kit (Affymetrix) and GeneChip Mouse Gene 1.0 ST array (Affymetrix). Signals were scanned with a GeneChip Scanner 3000 7 G, and data were analyzed using Affymetrix Expression Console software (Affymetrix). The robust multiarray average algorithm was used for log2 transformation and normalization of the GeneChip data. Hierarchical clustering analysis was performed using R (www.r-project.org). Functional enrichment analysis of selected genes was performed based on GO pathway annotation terms, with *P* values < 0.05 considered statistically significant.

#### Flow cytometric analysis, cell sorting, and DC culture

Single cells were prepared as described below. Briefly, tissues were collected from mice, cut into small pieces with scissors, and incubated in a collagenase solution (1 mg/ml collagenase and 0.04 mg/ml DNase I in PBS) for 30 min at 37 °C. The reaction was stopped by the addition of a stopping solution (10% FBS and 10 mM EDTA in PBS). Red blood cells were lysed with red blood cell lysis buffer (150 mM NH_4_Cl, 12.5 mM NaHCO_3_, and 0.1 mM EDTA). The cells were resuspended in FACS buffer (2% FBS in PBS), blocked with an anti-FcgRII/III antibody (2.4G10), and stained with primary antibodies (anti-CD11c, anti-MHC class II, anti-mouse BLT1, anti-CD80, anti-CD86, anti-B220, or anti-CD11b). The anti-mouse BLT1 antibody was visualized with SA-APC. Dead cells were stained with 7-AAD, and 7-AAD-negative (live) cells were analyzed. In some experiments, labeled splenic DCs were sorted using a FACSAria. For BMDC differentiation, bone marrow cells were collected from the femurs and tibias of C57BL/6 J mice with a syringe and 27 G needle. Cells (1 × 10^6^ cells/ml) were cultured for 6 days in DC medium (RPMI-1640 medium supplemented with 10% fetal bovine serum [FBS], 50 μM 2-mercaptoethanol, 10 ng/ml mouse GM-CSF, 100 U/ml penicillin, and 100 μg/ml streptomycin) for 6 days in 24-well plates (Sumilon, Tokyo, Japan) at 37 °C in an atmosphere of 5% CO_2_. The medium was changed every 2 days. On Day 4, floating cells were washed away. On Day 6, cells were stained and sorted using a FACSAria II flow cytometer (BD Biosciences).

### Immunofluorescence staining

Spleens were collected from C57BL/6 J mice and embedded in O.C.T. compound (Sakura Fintetek, Tokyo, Japan). Sections (5 μm thickness) were prepared using a cryostat (Leica, Wetzler, Germany). Tissue sections were fixed for 10 min in cold acetone, washed, and blocked for 30 min with 5% bovine serum albumin. The sections were incubated with primary antibodies specific for murine BLT1 (rabbit polyclonal antibody) and CD11c (FITC-conjugated antibody), followed by incubation with a biotin-conjugated anti-rabbit IgG antibody and SA-Alexa Fluor 594 in sequence. The stained sections were visualized with an Axiovert (Carl Zeiss, Gottingen, Germany).

### Mice and genotyping

**Fig. 2:**
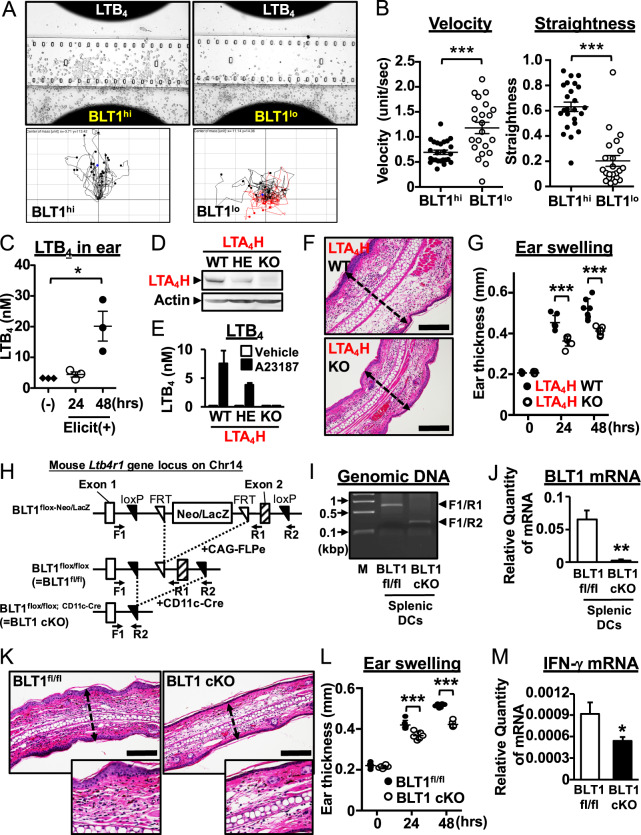
BLT1^hi^ DCs migrate toward LTB_4_ and accelerate dermatitis. **a**, **b** A chemotaxis assay was used to assess the migration of BLT1^hi^ DCs (*left*) and BLT1^lo^ DCs (*right*) derived from BMDCs toward LTB_4_. Cells were placed in the bottom chamber, and 100 nM LTB_4_ was added to the head chamber. Representative images are shown (**a**, *upper panels*). Tracking charts for each cell type are shown (*n* > 20). Black tracking lines indicate the cells moving forward, and red tracking lines indicate the cells moving backward (**a**, *bottom panels*). Velocity and straightness are shown in **b** (*n* > 20; error bars indicate the S.E.M.). **c** The fatty acid-enriched fraction from mouse ears was analyzed for LTB_4_ by LC-MS/MS. The time after elicitation is shown. (−): no elicitation. **d** Western blot analysis for the LTA_4_H protein was performed with BMDC protein lysates (WT wild-type, HE heterozygous, KO homozygous knockout). **e** The A23187 (calcium ionophore)-dependent production of LTB_4_ by BMDCs was analyzed by LC-MS/MS. WT wild-type, HE hetero, KO knockout. **f** H&E-stained ear sections from LTA_4_H WT and LTA_4_H KO mice are shown. Bars, 100 μm. Dotted lines indicate ear thickness. **g** Ear thickness was measured at 0, 24, and 48 h after elicitation in WT and LTA_4_H KO mice (*n* = 7). **h** The schematic shows the mouse ltb4r1 gene locus targeted to generate DC-conditional BLT1 knockout mice. **i** Genotyping was performed to evaluate the deletion of the targeted loci. The primer position is indicated in **h**. **j** The expression of BLT1 in splenic DCs was evaluated. **k**, **l**, **m** BLT1^fl/fl^ and BLT1 DC cKO mice were sensitized and challenged with the hapten DNFB. **k** H&E-stained ear sections from hapten-induced BLT1^fl/fl^ (*left panels*) and BLT1 DC cKO mice (*right panels*) are shown. Forty-eight hours after challenge, ear tissue was collected and stained. Bars, 100 μm. **l** Ear thickness was measured at 0, 24, and 48 h postelicitation in WT and BLT1 DC cKO mice (*n* = 6). **m** The mRNA expression of IFN-γ in ear tissue at 48 h postelicitation was measured by QPCR. β-actin was used as an internal control. *P < 0.05; ***P < 0.001; unpaired Student’s *t*-test (**b**, **j**, **m**); two-way ANOVA with Bonferroni’s *post hoc* tests (**c**, **g**, **l**)

C57BL/6 J mice were purchased from Japan SLC (Shizuoka, Japan). Ltb4r1^tm1a(EUCOMM)Hmgu^ embryonic stem (ES) cells (clone H11) were purchased from EUCOMM (ID: 45475) and used to generate chimeric mice *via* the aggregation method. Ltb4r1^tm1a(EUCOMM)Hmgu^ mice were crossed with CAG-FLPe Tg mice to remove the region containing the LacZ-neomycin cassette between the FRTs and then crossed with wild-type C57BL/6 J mice to remove the FLPe gene to generate BLT1^flox/flox^ mice. BLT1^flox/flox^ mice were then crossed with CD11c-Cre Tg mice. CD11c-Cre Tg mice and OT-II Tg mice were purchased from The Jackson Laboratory (Bar Harbor, ME, USA). LTA_4_H KO mice were generated in-house using a CRISPR/Cas9 system. Briefly, sgRNA and mRNA encoding Cas9 were microinjected into the cytoplasm of fertilized 1-cell eggs from C57BL/6 J female mice induced to undergo superovulation by intraperitoneal injection of PMSG followed by hCG at an interval of 48 h. These mice were then mated overnight with C57BL/6 J male mice. After microinjection, 2-cell embryos, which were cultured in modified Whitten’s medium for ~24 h and developed from the fertilized 1-cell eggs, were transferred into the oviducts of pseudopregnant ICR females (Charles River Laboratories Japan, Inc., Kanagawa, Japan). Mutations were evaluated by PCR, followed by a T7E1 enzyme assay and an Aat II enzyme assay (Fig. S4). Frameshift mutations were confirmed by sequencing. The sequence of the sgRNA used to generate LTA_4_H KO mice was 5′-CTCACTCCTGAACAGACGTCAGG-3′. Genomic DNA was extracted from LTA_4_H KO BMDCs using the DNeasy Blood & Tissue Kit (Qiagen, Hilden, Germany) according to the manufacturer’s instructions, and serial PCRs were performed before digestion with AatII. The PCR conditions were as follows: 94 °C for 30 s, 60 °C for 30 s, and 72 °C for 1 min, for 25 cycles (1^st^ PCR) or 35 cycles (2^nd^ PCR). The sequences of the primers used for genotyping LTA_4_H KO mice were as follows: LTA_4_H F1: 5′-TCACAGATGCAAAGTACGTGACACA-3′ and LTA_4_H R1: ACATGGCGGCCTTTGCTTA-3′; LTA_4_H F2: 5′-CTCACGCTGAACCGATGAGA-3′ and LTA_4_H R2: 5′-TTTCAGGGCCATTCACTGAGTC-3′. A FACSAria II flow cytometer (BD Biosciences, San Jose, CA, USA) was used to sort CD11c + /MHC class II + DCs from splenocytes isolated from BLT1^flox/flox^ and BLT1^flox/flox^:CD11c-Cre mice. Genomic DNA was prepared from splenic DCs and BMDCs using the DNeasy Blood & Tissue Kit (Qiagen). A KOD dash PCR kit (TOYOBO, Tokyo, Japan) was used to amplify the cDNA as follows: 94 °C for 20 s, 62 °C for 1 min, and 72 °C for 2 min (32 cycles). The sequences of the primers used for PCR were as follows: BLT1 Fw primer (F1): 5′-ACAGCCTGGTTAGGTTAGGAAATTAGTC-3′; BLT1 reverse primer 1 (R1): 5′-CACAGACAGTAGAACAATGGGCAACAG-3′; and BLT1 reverse primer 2 (R2): 5′-TAGGAACATAGGGCTATATCGG-3′. The positions of the primers are indicated in Fig. 2h. All animal experiments in this study were approved by the Ethics Committees for Animal Experiments of Juntendo University School of Medicine, Kyushu University and Kumamoto University and performed in accordance with approved guidelines and regulations.

### In vitro and in vivo T cell proliferation assays

BLT1^hi^ and BLT1^lo^ DCs were cocultured with CMFDA-labeled CD4^+^ T cells (ratio 1:5: 2 × 10^4^ DCs and 1 × 10^5^ CD4^+^ T cells) derived from the spleen of OT-II Tg mice. The OVA peptide was added to the cell culture medium and incubated for 3 days. CMFDA-positive CD4^+^ T cells were assessed by FACS analysis. For in vivo experiments, BLT1^hi^ and BLT1^lo^ DCs were cultured in vitro (for 24 h) with 1 μg/ml OVA peptide. Next, 3 × 10^6^ cells in 200 μL of saline were injected intravenously into OT-II Tg mice. Immediately after DC transfer, the mice received an intraperitoneal injection of 35 μg of CpG DNA in 250 μl of saline or saline alone (control). Three days after the DC transfer, the mice were sacrificed, and splenocytes were analyzed by flow cytometric analysis.

### Real-time quantitative RT-PCR (QPCR)

Total RNA was extracted from various murine tissues, including the ear, splenic DCs, and BMDCs, using TRIzol reagent. Reverse transcription (RT) was performed using a high-capacity cDNA reverse transcription kit (Thermo Fisher Scientific). The primer sequences used in this study were as follows: mouse BLT1 Fw: 5′-ATGGCTGCAAACACTACCATCTC-3′ and mouse BLT1 Rv: 5′-GACCGTGCGTTTCTGCATC-3′; mouse IL-12p35 Fw: 5′-AGACATCACACGGGACCAAAC-3′ and mouse IL-12p35 RV: 5′-CCAGGCAACTCTCGTTCTTGT-3′; mouse IL-12p40 FW: 5′-TGGTTTGCCATCGTTTTGCTG-3′ and mouse IL-12p40 RV: 5′-ACAGGTGAGGTTCACTGTTTCT-3′; mouse IL-2 FW: 5′-TGAGCAGGATGGAGAATTACAGG-3′ and mouse IL-2 RV: 5′-GTCCAAGTTCATCTTCTAGGCAC-3′; mouse IFN-γ Fw: 5′-ATGAACGCTACACACTGCATC-3′ and mouse IFN-γ Rv: 5′-CCATCCTTTTGCCAGTTCCTC-3′; mouse GAPDH FW: 5′-GTGGACCTCATGGCCTACAT-3′ and mouse GAPDH RV: 5′-GGGTGCAGCGAACTTTATTG-3′; and mouse β-actin Fw: 5′-CATCCGTAAAGACCTCTATGCCAAC-3′ and mouse β-actin Rv: 5′-ATGGAGCCACCGATCCACA-3′.

#### SDS-PAGE and western blot analysis

Microsomal fractions were prepared by serial centrifugation (at 800 × *g*, 10,000 × *g*, and 100,000 × *g*) of sonicated samples in sonication buffer (20 mM Tris-HCl [pH 7.4], 0.25 M sucrose, 10 mM MgCl_2_, and 2 mM EDTA-2Na). Whole-cell lysates were prepared in lysis buffer (25 mM HEPES [pH 7.4], 10 mM Na_4_P_2_O_7_, 100 mM NaF, 5 mM EDTA, 2 mM Na_3_VO_4_, and 1% Triton X-100) and separated on 10% SDS-PAGE gels. Proteins were then blotted onto a PVDF membrane and visualized using appropriate antibodies and ECL plus reagents (GE Healthcare, Princeton, NJ).

### Cytokine bead array

BLT1^hi^ and BLT1^lo^ DCs were stimulated with PAMPs (LPS or CpG DNA) for 24 h, and the culture medium was collected. Cytokine array analysis was performed using the BD CBA Assay Mouse Inflammation Kit (BD Biosciences) and Mouse Th1/Th2/Th17/Th22 13plex, TGF-β1, and IL-23 simplex (eBioscience) cytomixes. Bead array data were analyzed using a FACSCalibur flow cytometer (BD Biosciences).

### Delayed-type contact dermatitis and adoptive transfer

The mouse model of contact dermatitis used in this study was described previously.^36,37^ Briefly, mice were sensitized by application of 50 μl of 0.5% DNFB (in acetone/olive oil [4:1]) to a shaved abdominal site on Day 0, followed by application of 20 μl of 0.3% DNFB to the right ear on day 5. Ear thickness was measured using a digital micrometer (Mitutoyo, Tokyo, Japan). At 48 h postchallenge, the ears were collected and subjected to histological and QPCR analyses. For adoptive transfer, BLT1^hi^ and BLT1^lo^ DCs derived from BMDCs were sorted and loaded with 1 mM DNBS for 6 h in vitro. The hapten-loaded DCs were washed twice with PBS, and 5 × 10^5^ cells were injected into the footpad. Five days after DC transfer, the mice were challenged by application of 20 μl of 0.3% DNFB to the right ear.

### In vitro and in vivo chemotaxis assays

**Fig. 3:**
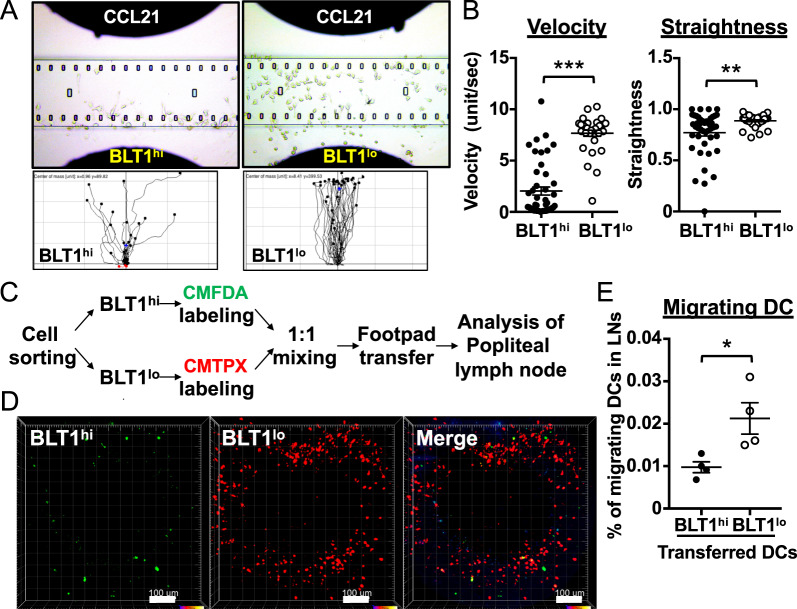
BLT1^lo^ DCs more robustly migrate toward CCL21 and lymph nodes than do BLT1^hi^ DCs. **a**, **b** Chemotaxis assay. The migration of BLT1^hi^ DCs (*left*) and BLT1^lo^ DCs (*right*) toward CCL21 was evaluated. Cells were placed in the bottom chamber, and 250 ng/ml CCL21 was added to the head chamber. Representative pictures are shown (**a**, *upper panels*). Tracking charts for each cell type are shown (*n* > 20). Black tracking lines indicate the cells moving forward, and red tracking lines indicate the cells moving backward (**a**, *bottom panels*). Velocity and straightness are shown in **b**. (*n* > 20, error bars indicate the S.E.M.) **c** Schematic of the experimental flow for DC transfer into the footpad. **d**, **e** In vivo migration assay. BLT1^hi^ and BLT1^lo^ DCs were stained with CMFDA and CMTPX, respectively. Stained DCs were mixed at a 1:1 ratio (2 × 10^6^ cells (**d**) and 2 × 10^5^ cells (**e**)) and injected into the footpad. The popliteal lymph nodes were assessed by two-photon microscopy (**d**) or flow cytometry (**e**) 24 h after DC transfer. Representative photos are shown. Bars indicate 100 μm

To assess the migration of DCs toward LTB_4_ and CCL21 in vitro, both DC subsets were added to the chamber of a TaxiScan-FL optical assay device (GE Healthcare). LTB_4_ (100 nM) or CCL21 (250 ng/ml) was added to the head chamber to generate a concentration gradient. Phase-contrast images were captured at 10-s intervals for 10 min (Fig. 2a, b) and 1-min intervals for 30 min (Fig. 3a, b) and imported as stacks into ImageJ (NIH, Bethesda, MD, USA). Velocity and straightness (referred to as “directionality”) were calculated for more than 20 migrating cells by manual tracking using the chemotaxis and migration tools in the add-in to ImageJ software. For in vivo migration assays, BLT1^hi^ and BLT1^lo^ DCs were sorted, incubated for 24 h to allow recovery, and then stained with 10 μM or 15 μM CMTPX, respectively.^38^ Both subsets were mixed at a 1:1 ratio (1 × 10^5^ cells/each) and injected into the footpad. Twenty-four hours later, the popliteal lymph nodes were collected, and lymph node cells were subjected to flow cytometric analysis.

### Two-photon imaging

BLT1^hi^ and BLT1^lo^ DCs were sorted, incubated for 24 h to allow recovery, and then stained with 10 μM or 15 μM CMTPX (Thermo Fisher Scientific), respectively.^38^ Both DC subsets were mixed at a 1:1 ratio (1 × 10^6^ cells/each) and injected into the footpad. Twenty-four hours later, the popliteal lymph nodes were isolated, maintained in medium bubbled with 95% O_2_/5% CO_2_ at 37 °C, and examined *via* two-photon microscopy using a modified protocol from a previous study.^39^ The imaging system comprised an upright two-photon microscope (TCS Sp5; Leica) equipped with a 20× water immersion objective (HCX APO: numerical aperture (NA), 1.0; Leica) and a femtosecond-pulsed infrared laser (Mai-Tai HP Ti:Sapphire: Spectra-Physics, Santa Clara, CA) tuned to 840 nm. Fluorescence was detected by an external nondescanned detector (NDD) with the following emission filters: 492/SP nm for the second harmonic generation (SHG), 525/50 nm for CMFDA, and 585/40 nm for CMTPX. Raw imaging data were processed using Imaris software (Bitplane, Oxford Instruments, Concord, MA).

### Statistical analysis

All results are presented as the mean ± SEM. Comparisons between two groups were performed using an unpaired *t*-test, and comparisons among multiple groups were performed by ANOVA with *post hoc* tests. The threshold for statistical significance was set at *p* < 0.05. All statistical analyses were performed using Prism software (GraphPad Software, San Diego, CA, USA).

## Results

### Identification of BLT1-positive and BLT1-negative DC subsets

To examine the role of BLT1 in DCs, we first analyzed the expression of BLT1 at the protein level. Due to the lack of a monoclonal antibody specific for mouse BLT1, the expression profile of BLT1 in specific leukocyte populations has been unclear. Recently, we generated a mouse monoclonal antibody specific for mouse BLT1 (7A8) by immunizing BLT1 KO mice with cells overexpressing mouse BLT1.^34^ Thus, we examined BLT1 expression in BMDCs and in DCs in several mouse tissues. Surprisingly, we found that BLT1 was expressed by ~30% of CD11c + /MHC class II + splenic DCs but not expressed by ~60% of this population (Fig. 1b). Cells showing one of these expression profiles were also observed in the BMDC and lung DC populations (Fig. 1c, f); however, no BLT1-expressing DCs were detected in the lymph nodes (Fig. 1g). We further evaluated BLT1-expressing DCs after removing F4/80-positive macrophages and Ly-6C-positive monocytes from the splenocyte population and confirmed that the removal of F4/80-positive macrophages and Ly-6C-positive monocytes did not affect the BLT1-positive and BLT1-negative DC subpopulations (Fig. S1). Immunofluorescence staining using a polyclonal antibody specific for mouse BLT1 identified two DC subsets in the mouse spleen (Fig. 1d). Next, we sorted these DC subsets from BMDCs and observed their morphologies after Diff-Quik staining. BLT1-expressing DCs (BLT1^hi^ DCs) had a round nucleus and thin dendrites, whereas BLT1-negative and low BLT1-expressing DCs (BLT1^lo^ DCs) had a relatively irregular nucleus, thick dendrites, and vacuoles (Fig. 1e). Collectively, these data suggest that there are two DC subsets that can be defined by the level of BLT1 expression: BLT1^hi^ DCs and BLT1^lo^ DCs.

### Characterization of BLT1^hi^ and BLT1^lo^ DC subsets by various cell-surface markers

Next, we examined the expression of cell-surface markers that characterize these DC subsets. BLT1^hi^ and BLT1^lo^ DCs expressed MHC class II and CD80 at similar levels; however, the expression of CD86 was higher in BLT1^hi^ DCs than in BLT1^lo^ DCs (Fig. S2). The mouse spleen harbors three major DC subsets (CD11b + CD8α- DCs, CD11b- CD8α + DCs, and B220 + plasmacytoid DCs [pDCs]).^40^ Therefore, we analyzed the relationships among these DC subsets within the BLT1^hi^ and BLT1^lo^ DC populations. Both the BLT1^hi^ and BLT1^lo^ DC subsets contained B220 + pDC and B220- conventional DC (cDC) populations (Fig. 1h, *upper panels*). Moreover, both DC subsets contained CD11b + DCs and CD11b- DCs in a similar ratio (Fig. 1h, *bottom panels*). These data suggest that BLT1^hi^ and BLT1^lo^ DCs exhibit similar patterns of cell-surface marker expression (and at similar levels) and that both subsets comprise known DC subpopulations including pDCs, CD11b + DCs, and CD11b − DCs (Fig. 1i).

### BLT1^hi^ DCs migrate toward LTB_4_ and accelerate dermatitis

We asked whether BLT1 plays a role in DC migration because BLT1 acts as an important chemotactic receptor in neutrophils and macrophages. First, we assessed the expression and function of LTB_4_. The data showed that BLT1^hi^ DCs highly expressed BLT1 at the mRNA and protein levels (Fig. S3A, B). We also found that LTB_4_ activated downstream signaling by ERK kinase only in BLT1^hi^ DCs, not in BLT1^lo^ DCs (Fig. S3C). We next analyzed the ability of both subsets to migrate toward LTB_4_. We found that BLT1^hi^ DCs preferentially migrated toward LTB_4_ (Fig. 2a, *upper* and *bottom*; Movies S1 and S2). Although BLT1^lo^ DCs migrated with a high velocity, the migration of BLT1^hi^ DCs was more direct than that of BLT1^lo^ DCs. This suggests that “random” migratory activity is high in BLT1^lo^ DCs but that “LTB_4_-dependent” migratory activity is high in BLT1^hi^ DCs (Fig. 2b). The data also indicate that the LTB_4_-BLT1 axis is required for the migration of BLT1^hi^ DCs to inflamed tissues in which the LTB_4_ concentration is highly increased (Fig. 2c). To further investigate whether the LTB_4_-dependent migration of BLT1^hi^ DCs is important in vivo, we generated LTA_4_H-deficient mice using a CRISPR/Cas9 system. LTA_4_H is a critical enzyme that converts LTA_4_ into LTB_4_ (Fig. 1a). The Aat II restriction enzyme site within exon 3 of the mouse LTA_4_H gene was mutated to introduce a frameshift mutation. This resulted in a band representing the cleaved LTA_4_H gene in WT bone marrow-derived DCs (BMDCs) and the uncleaved LTA_4_H gene in LTA_4_H KO BMDCs (Fig. S4). LTA_4_H deficiency was confirmed by western blot analysis of BMDC lysates (Fig. 2d). We confirmed that LTB_4_ production was abrogated in LTA_4_H KO BMDCs (Fig. 2e). Next, we examined the effects of LTA_4_H deficiency on a mouse model of allergic contact dermatitis. Ear swelling in LTA_4_H KO mice was ameliorated significantly (Fig. 2f, g). The infiltration of LTA_4_H KO mouse ear tissue by inflammatory cells was less severe than that in the ear tissue of WT mice (Fig. 2f). These results confirm that the LTB_4_-BLT1 axis is crucial for the aggravation of allergic dermatitis in vivo. To investigate the cell-specific role of the LTB_4_-BLT1 axis in DCs, we further generated DC-specific BLT1 cKO mice by crossing BLT1^flox/flox^ mice with CD11c-Cre transgenic (Tg) mice (Fig. 2h). As expected, splenic DCs and BMDCs from BLT1^flox/flox;CD11c-Cre^ (BLT1 cKO) mice harbored a genomic deletion in BLT1 (Figs. 2i and S5A, C). These cells also showed reduced mRNA expression of BLT1 (Figs. 2j and S5B). Next, we analyzed the effects of BLT1-deficient DCs on allergic dermatitis. As shown in Fig. 2k, l, ear swelling induced by DNFB was less severe in BLT1 cKO mice than in WT mice. In addition, the expression of IFN-γ in the ears of BLT1 cKO mice was suppressed (Fig. 2m). Taken together, these data suggest that the LTB_4_-BLT1 axis in DCs plays an important role in migration toward LTB_4_-enriched inflammatory areas and amplifies hapten-induced allergic contact dermatitis.

### BLT1^lo^ DCs migrate toward CCL21 and lymph nodes more than do BLT1^hi^ DCs

Because DC migration to the lymph nodes is crucial for allergic dermatitis, we investigated the migratory activity of both DC subsets in response to CCL21, a “homing” chemokine expressed in the lymph nodes. Surprisingly, BLT1^lo^ DCs showed greater migration toward CCL21 than did BLT1^hi^ DCs (Fig. 3a, *upper* and *bottom*; Movies S3 and S4). BLT1^lo^ DCs migrated more quickly and more directly than BLT1^hi^ DCs (Fig. 3b). We further asked whether BLT1^lo^ DCs migrate preferentially toward the lymph nodes in vivo. Sorted BLT1^hi^ DCs and BLT1^lo^ DCs were stained with 5-chloromethylfluorescein diacetate (CMFDA) and CMTPX, respectively, and then mixed at a 1:1 ratio. They were then injected into the footpad of naïve mice. Twenty-four hours later, more migrated BLT1^lo^ DCs were observed by 2-photon microscopy in the popliteal lymph nodes (Figs. 3c, d and S6; Movie S5). This preferential migratory activity of BLT1^lo^ DCs was also confirmed by flow cytometric analysis (Fig. 3e). These data suggest that BLT1^lo^ DCs preferentially migrate toward CCL21, which is enriched in the lymph nodes.

### Comparative analysis of the transcriptomic profiles of BLT1^hi^ and BLT1^lo^ DCs

**Fig. 4:**
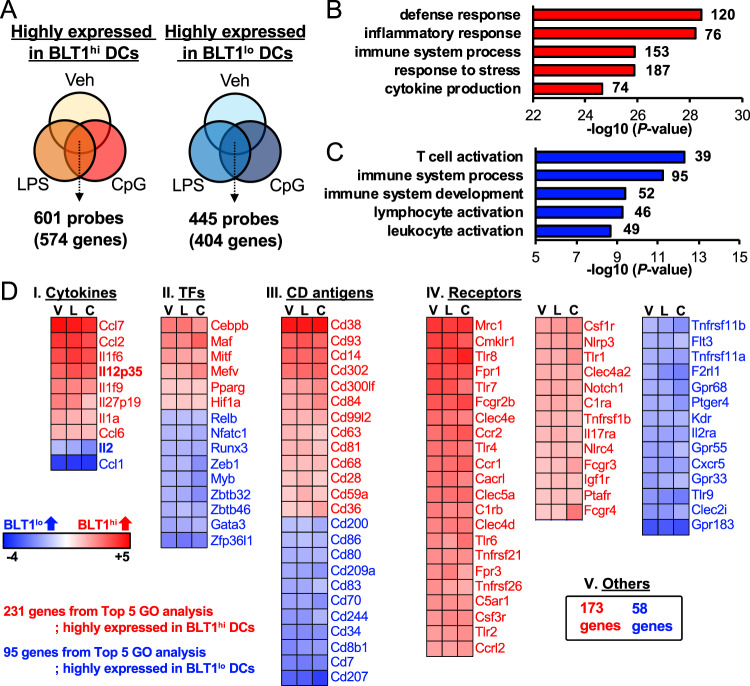
Comparative transcriptomic analysis of BLT1^hi^ and BLT1^lo^ DCs. **a** Venn diagrams show genes highly expressed in BLT1^hi^ DCs (*left*) and BLT1^lo^ DCs (*right*) under various conditions (exposure to LPS [100 ng/ml] and CpG DNA [1 μM] treatment for 4 h). Gene Ontology (GO) analysis was performed on 574 genes highly expressed in BLT1^hi^ DCs (**b**) and on 404 genes highly expressed in BLT1^lo^ DCs (**c**). The top 5 GO terms are listed. The numbers indicate the number of genes categorized in that GO term (574 genes (**b**) or 404 genes (**c**)). **d** The 231 genes listed in the top 5 GO terms for BLT1^hi^ DCs and the 95 genes listed in the top 5 GO terms for BLT1^lo^ DCs were categorized as follows: cytokines, transcription factors (TFs), CD antigens, receptors, and other. A heat map is shown. V, vehicle-treated group; L, LPS-treated group; C, CpG DNA-treated group

Next, we compared the transcriptomic profiles of BLT1^hi^ and BLT1^lo^ DCs with 207 profiles derived from granulocytes, B cells, NK cells, T cells, NKT cells, macrophages, monocytes, and DCs.^41^ The BLT1^hi^ and BLT1^lo^ DC subtypes localized next to each other within the DC cluster (Fig. S7). Next, we exposed both subsets to various PAMPs, including lipopolysaccharide (LPS) and CpG DNA, for 4 h and then performed microarray analysis. We extracted 601 probes (574 genes) highly expressed by BLT1^hi^ DCs and 445 probes (404 genes) highly expressed by BLT1^lo^ DCs under each condition from gene sets with high and common expression in both DC populations (Fig. 4a, Tables S1 and S2). The extracted genes were subjected to Gene Ontology (GO) analysis; the top 5 GO terms for the genes highly expressed by BLT1^hi^ DCs (*n* = 231) are shown in Fig. 4b, and those for the genes highly expressed by BLT1^lo^ DCs (*n* = 95) are shown in Fig. 4c. These genes (total 326 genes) were arranged in protein categories (Fig. 4d, Tables S3 and S4). Taken together, the data from the comparative transcriptome analysis suggest that BLT1^hi^ and BLT1^lo^ DCs are very similar but may differ in terms of their inflammatory defense responses, immune responses, and ability to activate T cells.

### BLT1^hi^ DCs preferentially induce Th1 differentiation by producing IL-12

**Fig. 5:**
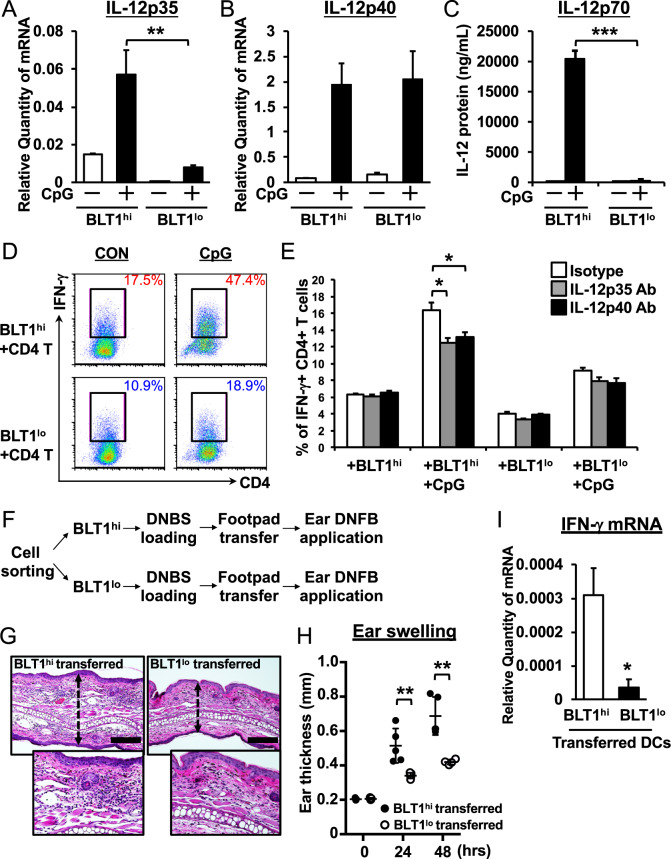
BLT1^hi^ DCs preferentially induce Th1 differentiation by producing IL-12p35. BLT1^hi^ DCs preferentially induce Th1 differentiation by producing IL-12p35. QPCR analysis of IL-12p35 (**a**) and IL-12p40 (**b**) expression was performed. Both DC subsets were treated with CpG DNA (500 nM) for 4 h. GAPDH was used as an internal control. **c** Both DC subsets were treated with CpG DNA for 24 h, and the supernatants were collected. The IL-12p70 protein in the supernatant was quantified by CBA analysis (*n* = 3; error bars indicate the S.E.M.). **d**, **e** Sorted BLT1^hi^ (*upper panels*) and BLT1^lo^ BMDCs (*bottom panels*) were cocultured for 3 days with splenic CD4+ T cells derived from OT-II Tg mice (ratio 1:4) in the presence (CpG) or absence (CON) of CpG DNA (500 nM). The cells were stained for intracellular IFN-γ and analyzed by flow cytometry. Cells were incubated for 3 days with neutralizing antibodies specific for IL-12p35 (1 μg/ml) or IL-12p40 (1 μg/ml) to examine the effects of IL-12 on Th1 differentiation. **f** The schematic shows the experimental flow for DC transfer into the footpad. **g**, **h**, **i** Both DC subsets were loaded with DNBS in vitro for 6 h and then injected into the footpad. Five days later, mouse ear tissue was treated with DNFB, and ear thickness was measured (**g**). Forty-eight hours after elicitation, ear tissue was collected and stained with H&E (**h**). Bars, 100 μm. **i** The mRNA expression of IFN-γ in induced mouse ear tissue was measured by QPCR. β-actin was used as an internal control (*n* = 3; error bars indicate the S.E.M.). *P < 0.05; **P < 0.01; ***P < 0.001; unpaired Student’s *t*-test (**i**); one-way ANOVA with Bonferroni’s *post hoc* tests (**a**, **b**, **c**, **e**); two-way ANOVA with Bonferroni’s *post hoc* test (**h**)

Unbiased microarray analysis demonstrated that BLT1^hi^ DCs expressed high levels of IL-12p35 (Fig. 4d). IL-12, which induces Th1 differentiation, comprises IL-12p35 and IL-12p40. First, we checked the expression of IL-12p35 and IL-12p40 by BLT1^hi^ and BLT1^lo^ DCs. Consistent with the microarray data, BLT1^hi^ DCs expressed high levels of IL-12p35; however, the subsets expressed IL-12p40 at the same level (Fig. 5a, b). Furthermore, only BLT1^hi^ DCs generated a heterodimeric IL-12p70 protein (Fig. 5c). Next, we used a coculture system to examine Th1 induction by both DC subsets. CD4+ T cells from OT-II Tg mice were cocultured with each DC subset in the presence of an ovalbumin (OVA) peptide. We found that CD4+ T cells preferentially differentiated into interferon (IFN)-γ positive Th1 cells when cocultured with BLT1^hi^ DCs (Fig. 5d). The addition of CpG DNA to the coculture system enhanced IFN-γ production, especially in BLT1^hi^ DC cultures. This BLT1^hi^ DC-dependent IFN-γ production was blocked with neutralizing antibodies specific for IL-12p35 or IL-12p40 (Fig. 5e). Next, we used a delayed-type contact dermatitis model generated by the transfer of antigen-loaded DCs into naïve mice to analyze the in vivo functions of both DC subsets. Sorted BLT1^hi^ or BLT1^lo^ DCs were loaded with sodium 2,4-dinitrobenzenesulfonate (DNBS) for 6 h and then injected into the footpad of naïve mice. Five days later, the mouse ears were treated with 2,4-dinitrofluorobenzene (DNFB) (Fig. 5f). At 48 h posttreatment, the ears of the mice receiving BLT1^hi^ DCs were thicker than those of the mice receiving BLT1^lo^ DCs (Fig. 5g, h). Moreover, IFN-γ was expressed at higher levels in the ears of the BLT1^hi^ DC-transferred mice than in those of the BLT1^lo^ DC-transferred mice (Fig. 5i). Taken together, these data suggest that BLT1^hi^ DCs induce Th1-dependent allergic contact dermatitis *via* specific production of IL-12 both in vitro and in vivo.

### BLT1^lo^ DCs preferentially induce T cell proliferation by producing IL-2

**Fig. 6:**
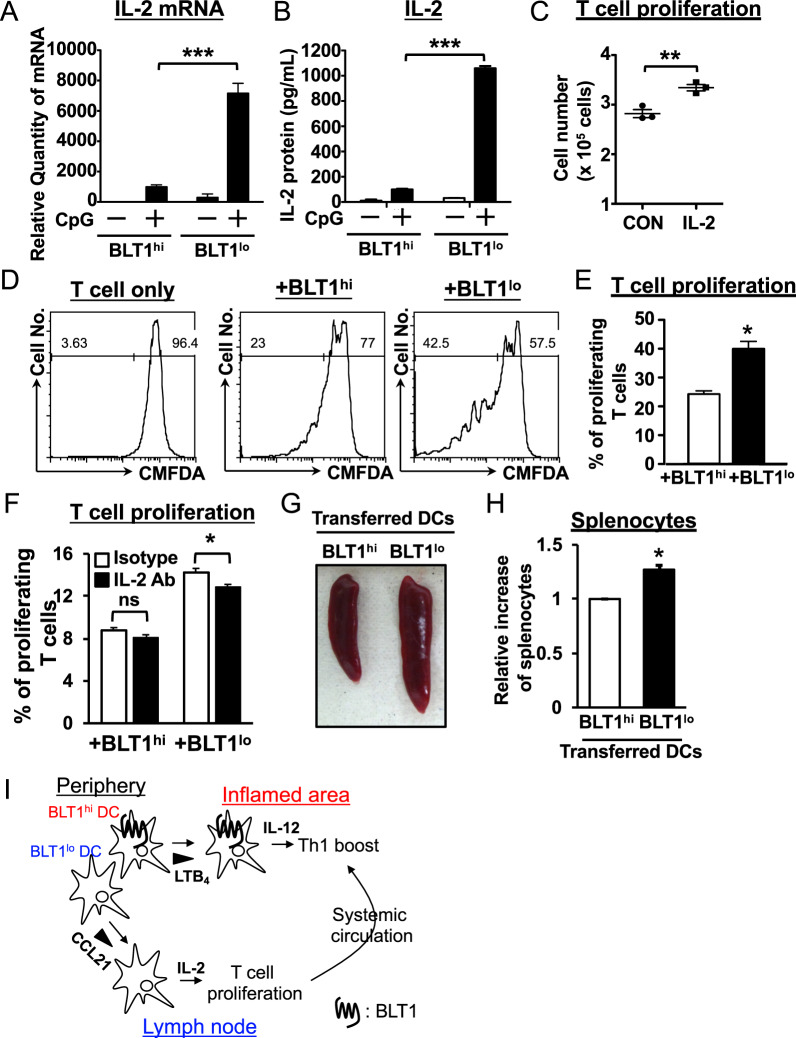
BLT1^lo^ DCs preferentially induce T cell proliferation by producing IL-2. **a**, **b** BLT1^hi^ and BLT1^lo^ DCs were treated with CpG DNA (500 nM) for 4 h and 24 h, followed by QPCR and CBA analyses for IL-2, respectively. **c** CD4+ T cells were cultured for 3 days with a recombinant IL-2 protein (10 ng/ml) or vehicle (CON), and cell numbers were counted. CD4+ T cells derived from the OT-II Tg spleen were stained with CMFDA and cocultured for 3 days (**d**, **e**) and 2 days (**f**) with either BLT1^hi^ or BLT1^lo^ DCs in the presence of an OVA peptide. Proliferating CD4+ T cells were analyzed by flow cytometric analysis (*n* = 3; error bars indicate the S.E.M.). A neutralizing antibody against murine IL-2 was added to the coculture system in **f** (10 μg/ml). A rat IgG2a kappa isotype control antibody was added to the control group. **g**, **h** BLT1^hi^ and BLT1^lo^ DCs were loaded with the OVA peptide (1 μg/ml) for 16 h and injected intravenously into OT-II Tg mice. CpG DNA was also injected intraperitoneally. At three days post-DC transfer, spleen size and splenocyte number were evaluated (*n* = 2; error bars indicate the S.E.M.). *P < 0.05; **P < 0.01; ***P < 0.001; unpaired Student’s *t*-test (**c**, **e**, **g**); one-way ANOVA with Bonferroni’s *post hoc* test (**a**, **b**). **i** The schematic model shows how the novel BLT1^hi^ and BLT1^lo^ DC subsets control skin inflammation. There are two DC subsets, BLT1^hi^ and BLT1^lo^ DCs, in peripheral tissues. BLT1^lo^ DCs migrate preferentially toward draining lymph nodes and produce high amounts of IL-2 to induce T cell proliferation. On the other hand, BLT1^hi^ DCs migrate toward LTB_4_, which is produced by inflammatory cells, such as neutrophils, in inflammatory areas. BLT1^hi^ DCs produce large amounts of IL-12, which boosts Th1 differentiation. Expanded differentiated Th1 cells produce IFN-γ, which drives spongiosis and edema in inflamed peripheral tissues

Next, we examined the specific function of BLT1^lo^ DCs. First, we reanalyzed the microarray data and found that BLT1^lo^ DCs expressed high levels of IL-2 (Fig. 4d). Because DC-derived IL-2 is crucial for the initiation of T cell proliferation,^42^ we next examined the expression of IL-2 by both DC subsets. As expected, we found that BLT1^lo^ DCs expressed high mRNA and protein levels of IL-2 (Fig. 6a, b). IL-2 supported the proliferation of splenic CD4+ T cells derived from OT-II Tg mice (Fig. 6c). Next, we used a coculture system to examine T cell proliferation induced by both DCs. When CD4+ T cells from OT-II Tg mice were cultured with BLT1^lo^ DCs in the presence of an OVA peptide, they proliferated much faster than when cocultured with BLT1^hi^ DCs plus the peptide (Fig. 6d, e). This BLT1^lo^ DC-dependent CD4+ T cell proliferation was significantly blocked with a neutralizing antibody specific for IL-2 (Fig. 6f). Therefore, we asked whether BLT1^lo^ DCs have a much greater potential to induce antigen-dependent T cell proliferation in vivo. OVA peptide-loaded DCs were injected intravenously into OT-II Tg mice. The spleen was then removed to examine antigen-dependent T cell proliferation. We observed splenomegaly and increased splenocyte numbers only in the mice receiving BLT1^lo^ DCs (Fig. 6g, h). Collectively, these data suggest that BLT1^lo^ DCs have a greater potential to induce T cell proliferation than do BLT1^hi^ DCs, likely *via* specific production of IL-2. Collectively, our data demonstrate that BLT1^lo^ DCs produce IL-2 to drive T cell proliferation after migrating to CCL21-rich lymph nodes, whereas BLT1^hi^ DCs migrate toward peripheral LTB_4_-rich inflammatory areas to produce IL-12 to drive peripheral Th1 differentiation to deliver a “final boost” to tightly control Th1-dependent immune responses (Fig. 6i).

## Discussion

BLT1, which is expressed by neutrophils, eosinophils, macrophages, DCs, CD4+ T cells, and CD8+ T cells, plays roles in innate and acquired immune responses and several immunological disorders. Previously, we and others have reported that systemic BLT1 deficiency ameliorates Th1-dependent contact dermatitis, Th2-dependent asthma, Th17-dependent EAE, and rheumatoid arthritis.^26,28,31,32,43–45^ Despite its importance in immunity, the cell-specific role of BLT1 is largely unknown due to the lack of cell-specific BLT1 cKO mice and specific monoclonal antibodies. Here, we generated DC-specific BLT1 cKO mice. A BLT1 cKO mouse model of allergic contact dermatitis showed decreased ear swelling and IFN-γ expression, clearly suggesting that BLT1 expression by DCs plays a crucial role in Th1-dependent skin inflammation (Fig. 6i). A growing body of evidence shows that lipid mediators, including LTB_4_, PGs, resolvins, and sphingolipids (and their cognate receptors), act as crucial immunomodulators. Among these factors, the S1P receptor (S1P_1_) is a target for the treatment of multiple sclerosis.^7,8^ In addition, antagonists of CysLT1, which is a receptor for LTC_4_ and LTD_4_, are widely used to treat asthma.^46,47^ Although lipid mediators and their receptors are important therapeutic targets in various immunological disorders, there is no drug that targets the LTB_4_-BLT1 axis.^17^ Here, we identified an important cell-specific immunological function of the LTB_4_-BLT1 axis in vitro and in vivo, clearly suggesting that BLT1 may be a novel drug target for immunological disorders, such as allergic dermatitis. In addition, we recently solved the X-ray structure of BLT1 and its antagonist BIIL260 at 3.7-Å resolution.^48^ These works will accelerate the structure-based design of novel drugs that target BLT1.

Here, we identified two distinct DC subsets: BLT1^hi^ DCs and BLT1^lo^ DCs. Numerous reports have shown that BLT1 is expressed by various leukocytes at the mRNA level. However, the expression of the BLT1 protein by specific cell types has been unclear due to the lack of antibodies specific for mouse BLT1. Recently, we created an anti-mouse BLT1 monoclonal antibody^34^ and used it to identify novel DC subsets within splenic DC, BMDC, and lung DC (but not lymph node DC) populations, although previous reports (including ours) indicated that BLT1 was expressed by DCs only at the mRNA level. Here, we found that both BLT1^hi^ DCs and BLT1^lo^ DCs showed a similar phenotype with respect to the expression of cell-surface proteins such as MHC class II, CD80, CD86, B220, and CD11b (BLT1 is the exception). However, comparative transcriptomic profiling revealed that BLT1^hi^ DCs expressed high levels of IL-12, whereas BLT1^lo^ DCs expressed high levels of IL-2, resulting in distinct functions (i.e., preferential effects on Th1 differentiation and T cell proliferation, respectively, in vitro and in vivo). At present, we do not know how these distinct cytokine profiles are generated. We also concluded that LTB_4_-BLT1 signaling is unlikely to be the reason for the difference (Fig. S8). Thus, other as-yet-unidentified mechanisms may exist. One possibility is that intracellular signaling cascades differ between BLT1^hi^ and BLT1^lo^ DCs. Our in silico analysis using the Chromatin Immunoprecipitation (ChIP)-Atlas (http://chip-atlas.org) revealed that the transcription factors regulating IL-12p35 and IL-2 are different (i.e., interferon regulatory factor 1 [IRF1], which regulates IL-12p35, and CEBP-β, which regulates IL-2, are localized within acetylated H3- and H3K4me1-enriched enhancer regions on each gene locus) (Fig. S9). In addition, although we used the same TLR9 ligand (CpG DNA), we found that the activation of intracellular signaling pathways in BLT1^hi^ and BLT1^lo^ DCs was different. These data lead us to hypothesize that the signaling downstream of TLR9 differs between BLT1^hi^ and BLT1^lo^ DCs, resulting in the activation of different transcription factors and induction of different cytokines. Another possibility is that the epigenetic status of each DC subset differs. The microarray data showed differential expression of several epigenetics-modifying enzymes in BLT1^hi^ and BLT1^lo^ DCs. In particular, BLT1^hi^ DCs express high levels of a histone demethylase (Jumonji Domain-containing Histone Demethylase [Jhdm] 1D) and DNA demethylase (Ten-Eleven Translocation [TET] 1), whereas BLT1^lo^ DCs express high levels of a different histone demethylase, Jhdm1b (Tables S1 and S2). Importantly, BLT1, IL-12p35, IL-12p40, and IL-2 are regulated either directly or indirectly by epigenetic mechanisms.^49–55^ In a future study, we will focus on the molecular mechanisms underlying the differential regulation of cytokine expression in BLT1^hi^ and BLT1^lo^ DCs at the intracellular signaling and/or epigenetic levels.

Another interesting finding was that BLT1^hi^ DCs preferentially migrated toward LTB_4_, which is produced in inflamed peripheral areas (Fig. 2) rather than the lymph nodes. In contrast, BLT1^lo^ DCs exhibited preferential migration toward CCL21, which is produced in the lymph nodes (Fig. 3). These data suggest that BLT1^hi^ and BLT1^lo^ DCs have different migratory patterns. The fundamental function of DCs is to capture antigens and migrate to draining lymph nodes. However, recent reports have shown that DC migration toward peripheral areas is crucial for fine-tuning immune responses.^2–4,56^ Migrated DCs form clusters and drive peripheral T cell expansion and B cell-mediated immunity; thus, DC migration to the lymph nodes and inflamed peripheral areas is important for regulating acquired immunity. These reports lead us to hypothesize that the formerly identified canonical DCs are in fact BLT1^lo^ DCs and the latter noncanonical DCs are in fact BLT1^hi^ DCs. In the future, we will investigate the possibility that BLT1^hi^ DCs contribute to the formation of inducible peripheral lymphoid clusters, including inducible skin-associated lymphoid tissue (iSALT) and inducible bronchus-associated lymphoid tissue (iBALT). One area that remains to be explored is the mechanism underlying the differences in the motility of these DCs. Although the expression of the mRNA transcript encoding C-C chemokine receptor type 7 (CCR7), a receptor for CCL21, was comparable between BLT1^hi^ and BLT1^lo^ DCs, the population of CCR7-positive cells was higher in bone marrow-derived and splenic BLT1^lo^ DCs (Fig. S10). This difference could, at least in part, explain the distinct migratory activities of both DC subsets. In addition, we speculate that mechanisms other than the CCL21/CCR7 axis may be involved. One possible hypothesis is that the PGE_2_-EP4 axis is highly active in BLT1^lo^ DCs, resulting in preferential migration of BLT1^lo^ DCs toward the lymph nodes. The PGE_2_-EP2/EP4 axis was shown to accelerate DC migration toward the lymph nodes in both mice and humans through intracellular signaling cross-talk and matrix metalloproteinase upregulation without alterations in the cell-surface expression of CCR7.^57–60^ The importance of the PGE_2_-EP4 axis in DC migration is also highlighted in the report by Kabashima et al., which showed impaired migration of Langerhans cells and reduced skin immune responses in EP4 KO mice.^61^ Our microarray data showed that BLT1^lo^ DCs expressed higher levels of EP4 (*Ptger4*) mRNA than BLT1^hi^ DCs; moreover, BLT1^lo^ DCs produced more PGE_2_ than BLT1^hi^ DCs (data not shown). CCL21 drives CCR7-dependent increases in the levels of intracellular Ca^2+^, which generates PGE_2_ and results in increased cell migration *via* signaling by the EP4 receptor, which is expressed at high levels on BLT1^lo^ DCs. The contribution of the PGE_2_-EP4 axis to the preferential migration of BLT1^lo^ DCs toward CCL21 and/or the lymph nodes will be investigated in a future study.

In summary, we generated LTA_4_H-deficient mice and BLT1 cKO mice and used them to show that BLT1 intensifies DC-mediated immune responses. We also identified novel DC subsets, which were defined by differential expression of BLT1 (high and low). These subsets show distinct cytokine-producing and migratory profiles. The data provide new insight into our understanding of the role of BLT1 in immune responses and suggest a potential therapeutic target in immunological disorders, including allergic contact dermatitis.

## Supplementary information

Supplementary Figure S1 to S10

Supplementary Table S1

Supplementary Table S2

Supplementary Table S3

Supplementary Table S4

Supplementary Movie S1

Supplementary Movie S2

Supplementary Movie S3

Supplementary Movie S4

Supplementary Movie S5
