# Supplementary material for: Expression of leukotriene B4 receptor 1 defines functionally distinct DCs that control allergic skin inflammation
Source: Cell Mol Immunol. 2020 Oct 9;18(6):1437–49. doi: 10.1038/s41423-020-00559-7 (PMC8167169; doi:10.1038/s41423-020-00559-7)
Supplement: Supplementary file 1 — Supplementary Figure S1 to S10 [file 41423_2020_559_MOESM1_ESM.pdf]

Supplementary Information

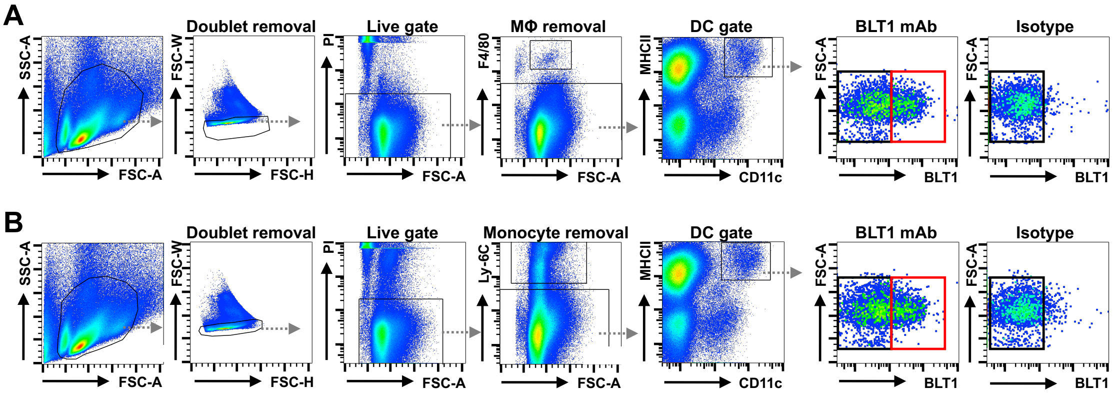

Figure S1. BLT1-expressing DCs exist in both macrophage and monocyte depleted CD11c+/MHC class II+ DC populations.

A. Splenic BLT1^{hi} and BLT1^{lo} DCs were evaluated in F4/80+ macrophage-depleted CD11c+/MHC class II+ DC populations. B. Splenic BLT1^{hi} and BLT1^{lo} DCs were evaluated in Ly-6C+ monocyte-depleted CD11c+/MHC class II+ DC populations. Red square: BLT1^{hi} DCs; Black square: BLT1^{lo} DCs.

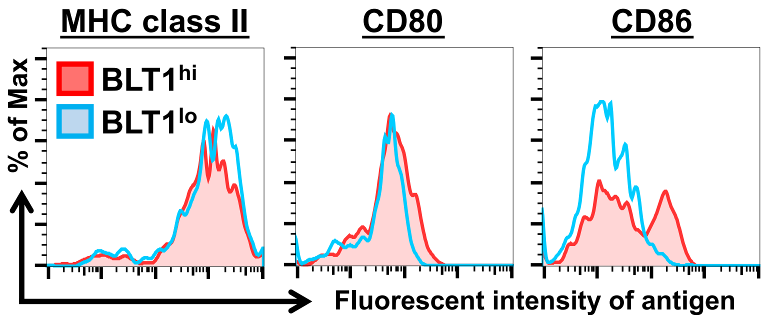

Figure S2. Comparative analysis of cell surface antigens between BLT1^{hi} and BLT1^{lo} DCs.

Splenic BLT1^{hi} and BLT1^{lo} DCs were stained with antibodies specific for MHC class II, CD80, and CD86. Red line: BLT1^{hi} DCs; Blue line: BLT1^{lo} DCs.

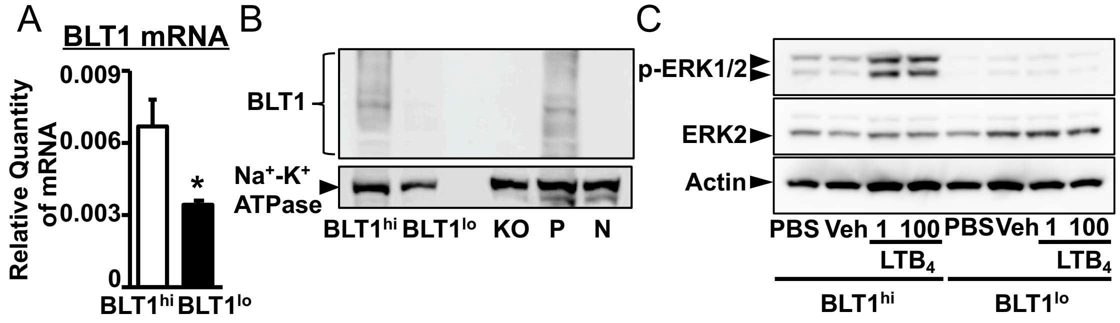

Figure S3. BLT1^{hi} DCs but not in BLT1^{lo} DCs express BLT1 at mRNA, protein and functional levels. **(A)** Total RNA was extracted from sorted BMDC-derived BLT1^{hi} and BLT1^{lo} DCs and subjected to QPCR analysis. GAPDH was used as an internal control (n=4; Error bars indicate the S.E.M.). *, $P < 0.05$; unpaired Student's *t*-test. **(B)** The microsomal fraction was extracted from BM-derived BLT1^{hi} and BLT1^{lo} DCs, and subjected to western blot analysis. Na+-K+ ATPase was used as a loading control. KO, microsomal fraction from BMDCs derived from BLT1 systemic knockout mice. P, positive control (microsomal fraction from CHO cells stably-expressing mouse BLT1). N, negative control (microsomal fraction from CHO cells). **(C)** Sorted BLT1^{hi} and BLT1^{lo} DCs were treated with 1 and 100 nM LTB$_4$. Thirty minutes after the stimulation, proteins were extracted and subjected to western blotting to detect phosphorylated ERK1/2 and pan-ERK2. Actin was used as a loading control.

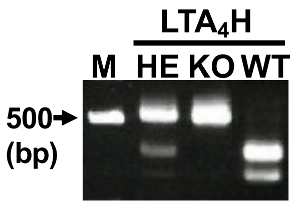

Figure S4. Generation of LTA_4H knockout mouse by CRISPR/Cas9 system. Recognition site of AatII was abrogated by CRISPR/Cas9. Genomic DNA from mouse tail was used for PCR and AatII enzyme cut. Deletion of AatII site was confirmed in KO strains.

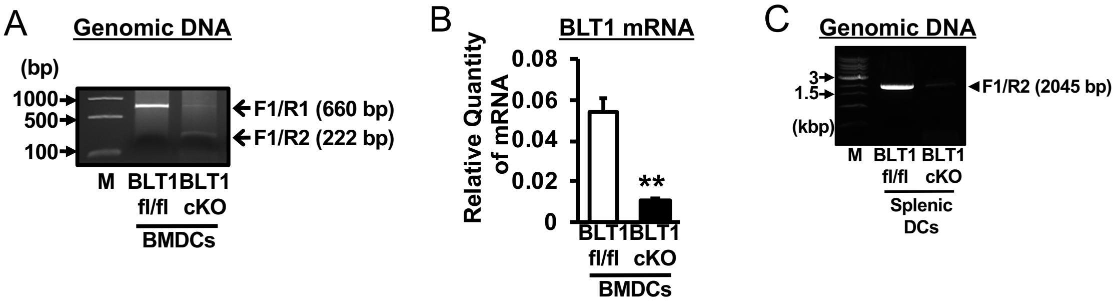

Figure S5. **Generation of DC-specific BLT1 conditional knockout mice. (A)** Genomic DNA from BMDCs was used for genotyping. Primer position was indicated Fig. 2H. **(B)** Cell-specific BLT1 knockout was confirmed by QPCR analysis of total RNA extracted from BMDCs. GAPDH was used as an internal control. **, $P < 0.01$. **(C)** Genomic DNA from splenic DCs was used for genotyping. Primer position was indicated Fig. 2H.

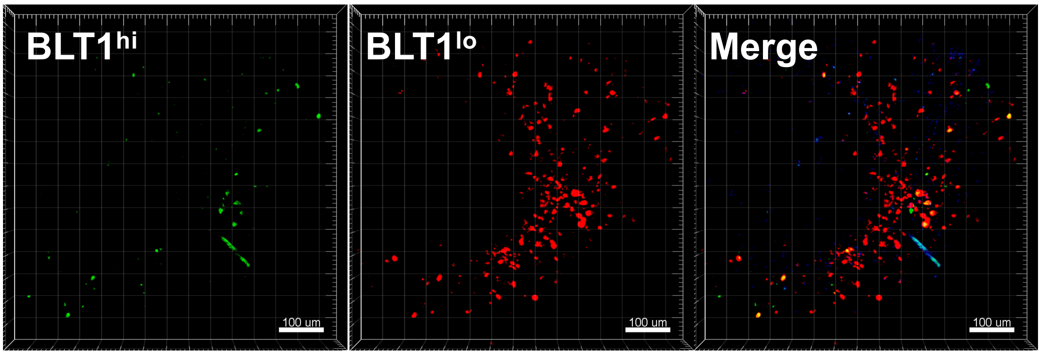

Figure S6. Migration of BLT1^{hi} and BLT1^{lo} DCs *in vivo* in another experiment. Sorted BLT1^{hi} and BLT1^{lo} DCs were stained with CMFDA and CMTPX, respectively. Stained DCs were mixed at a 1:1 ratio (total cell numbers: 2 x 10^6 cells) and injected into the footpad. Popliteal lymph nodes were assessed by two-photon microscopy 24 hours after DC transfer. Representative photos are shown. Bars indicate 100 μm.

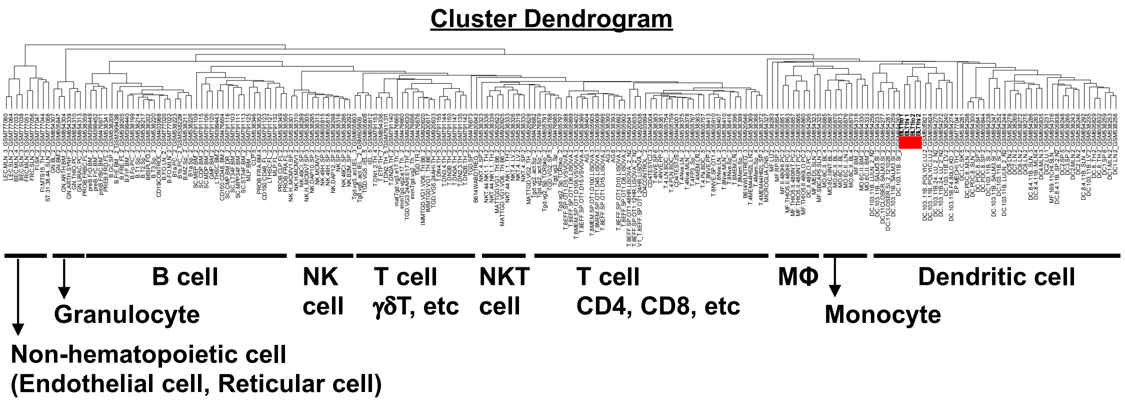

Figure S7. Comparative transcriptome analysis of BLT1^{hi} and BLT1^{lo} DCs. Cluster dendrogram showing transcriptome profiling of 209 types of hematopoietic and non-hematopoietic cells, including BLT1^{hi} and BLT1^{lo} DCs. Gene sets (772 probes) showing differences in signal values (-fold change > 128) among cells, were used for cluster analysis. Red line indicates BLT1^{hi} DCs (BLT1hi 1: BLT1^{hi} DCs after sorting; BLT1hi 2: BLT1^{hi} DCs incubated in DC medium for 4 hours) and BLT1^{lo} DCs (BLT1lo 1; BLT1^{lo} DCs after sorting; BLT1lo 2; BLT1^{lo} DCs incubated in DC medium for 4 hours).

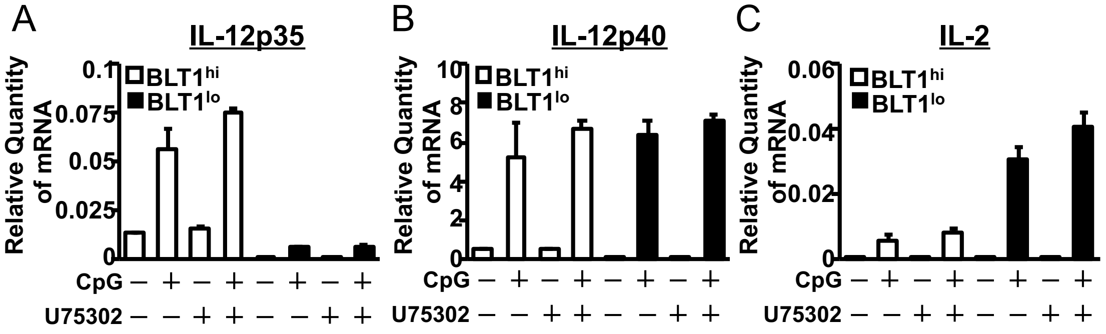

Figure S8. The LTB$_4$-BLT1 axis does not affect distinct cytokine expression in BLT1^{hi} and BLT1^{lo} DCs. (A, B, C) BLT1^{hi} and BLT1^{lo} DCs were pretreated for 30 min with the BLT1 antagonist U75302 (1 μM) followed by CpG DNA (500 nM) for 4 hours. Expression of IL-12p35 (A), IL-12p40 (B), and IL-2 mRNA (C) was measured by QPCR (n=3; Error bars indicate the S.E.M.).

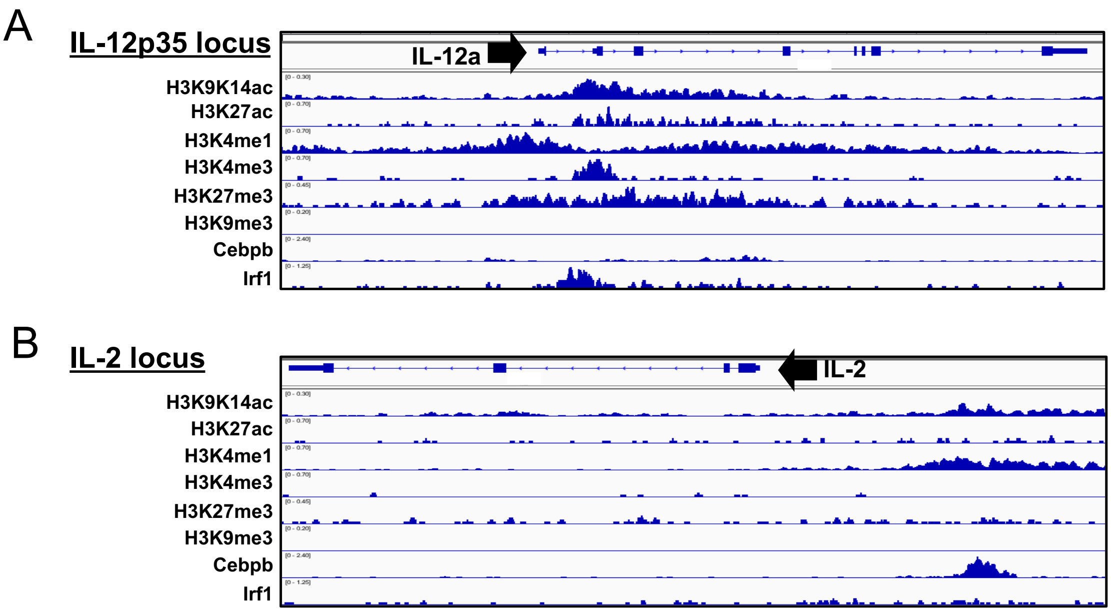

Figure S9. Several histone modifications (H3K9K14ac, H3K27ac, H3K4me1, H3K4me3, H3K27me3, and H3K9me3) and transcription factor bindings (Cebpb and Irf1) to the IL-12p35 (A), and IL-2 (B) loci in mouse dendritic cells. Data were obtained by ChIP-Atlas and visualized with IGV viewer. Arrows indicate the direction of transcription. H3 acetylation is a mark for actively transcribed region. H3K4me1 is a mark of enhancer region. H3K4me3 is a promoter mark. Interferon regulatory factor (IRF) 1 localizes in promoter and enhancer/promoter boundary of IL-12a locus, whereas Cebpb localizes in enhancer region of IL-2 locus.

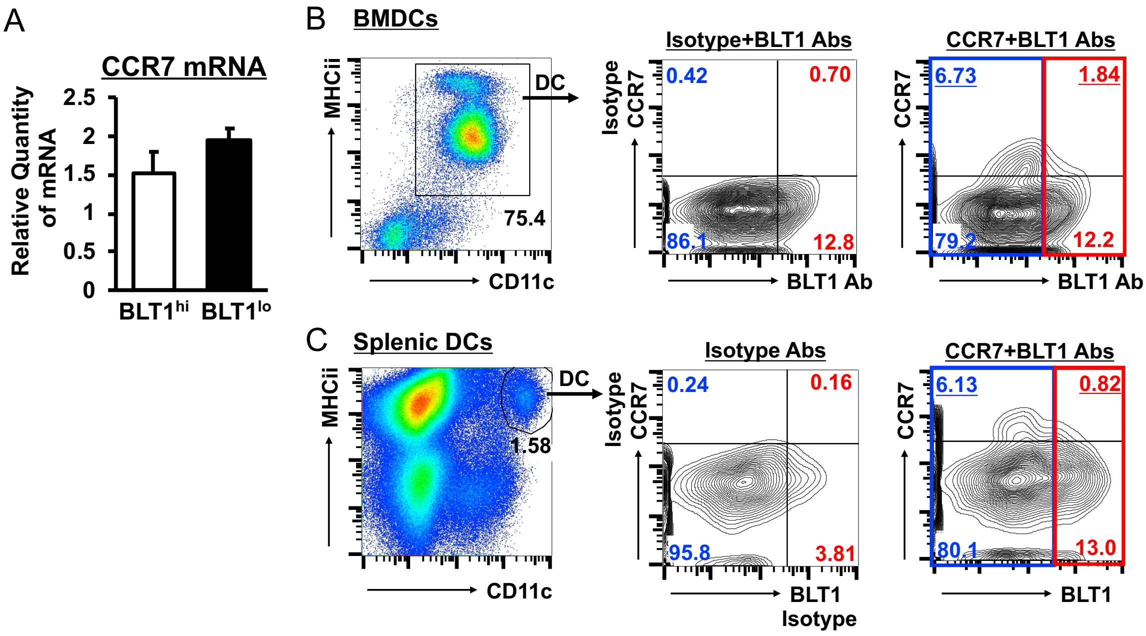

Figure S10. Expression of CCR7 mRNA and protein by BLT1^{hi} and BLT1^{lo} DCs. **(A)** Total RNA was extracted from both DC subsets and assessed by QPCR analysis. GAPDH was used as an internal control. **(B, C)** BMDCs (B) or splenic DCs (C) were stained with CD11c, MHC class II, BLT1 and CCR7 antibodies.
